# Efficacy of Electromyographic-Biofeedback Supplementation Training with Patellar Taping on Quadriceps Strengthening in Patellofemoral Pain Syndrome among Young Adult Male Athletes

**DOI:** 10.3390/ijerph18094514

**Published:** 2021-04-23

**Authors:** Asma Alonazi, Shahnaz Hasan, Shahnawaz Anwer, Azfar Jamal, Suhel Parvez, Faiz Abdulaziz Saleh Alfaiz, Heng Li

**Affiliations:** 1 Department of Physiotherapy, College of Applied Medical Sciences, Majmaah University, Majmaah 11952, Saudi Arabia; a.alonazi@mu.edu.sa; 2 Department of Building and Real Estate, The Hong Kong Polytechnic University, Hung Hom, Hong Kong; shahnawaz.anwer@connect.polyu.hk (S.A.); heng.li@polyu.edu.hk (H.L.); 3 Health and Basic Science Research Centre, Majmaah University, Majmaah 11952, Saudi Arabia; azfarjamal@mu.edu.sa; 4 Department of Biology, College of Science, Al-Zulfi, Majmaah University, Majmaah 11952, Saudi Arabia; f.alfaiz@mu.edu.sa; 5 Department of Medical Elementology and Toxicology, Jamia Hamdard, New Delhi 110062, India; sparvez@jamiahamdard.ac.in

**Keywords:** pain, strength, function, PFPS, athletes, young adults

## Abstract

This study compares the effects of electromyographic-biofeedback (EMG-BF)-guided isometric quadriceps strengthening with patellar taping and isometric exercise alone in patellofemoral pain syndrome (PFPS) among young adult male athletes. Sixty young adult male athletes with PFPS participated in the study. Participants were randomly divided into two groups: (1) EMG-BF-guided isometric exercise training with patellar taping (experimental group, *n* = 30), and (2) sham EMG-BF training with an isometric exercise program (control group, *n* = 30). Participants conducted their respective exercise programs for five days per week across four weeks. Study outcomes were pain (measured by the visual analog scale), functional disability (measured by the Kujala Anterior Knee Pain scale), and quadriceps strength (measured by an ISOMOVE dynamometer). Measurements were taken at baseline, Week 2, Week 4, and during a follow-up at Week 6. The experimental group demonstrated significantly lower VAS score at Weeks 2 and 4 compared to that of the control group (*p* = 0.008 and 0.0005, respectively). The score remained significantly lower at the Week 6 follow-up compared to the control group (*p* = 0.0005). There were no differences in knee function at Weeks 2 and 4 between the two groups (*p* = 0.086 and 0.171, respectively); however, the experimental group showed significantly better knee function at Week 6 compared to the control group (*p* = 0.002). There were no differences in quadriceps strength at Week 2 between the two groups (*p* = 0.259); however, the experimental group demonstrated significantly higher quadriceps strength at Weeks 4 and 6 compared to the control group (*p* = 0.0008). Four weeks of EMG-BF supplementation training with patellar taping demonstrated significant improvements in pain intensity, functional disability, and quadriceps muscle strength in young adult male athletes with PFPS.

## 1. Introduction

Patellofemoral pain syndrome (PFPS) is a common musculoskeletal disorder in the general population, predominantly in adolescents and young athletes [1,2,3,4]. Several reports suggested that about 25–30% of sports-related injuries [5,6] and up to 40% of knee disorders [4,7,8] are associated with PFPS. Other studies suggested that the prevalence of PFPS in female and male athletes was about 33% and 18%, respectively [9,10]. Additionally, PFPS is considered to be one of the most common overuse injuries in other sports including basketball [11,12], volleyball [13,14], and running [14,15,16,17].

PFPS has many possible causes that are usually due to problems in biomechanics such as weakness of the vastus medialis obliquus (VMO), resulting in abnormal patellar tracking between the patella and the femur [18]. Under normal circumstances, the patella presses against the lower portion of the femur when the knee is in a bent position [19,20]. It is caused by imbalances in the forces controlling patellar tracking during knee flexion and extension, particularly in the knee joint. PFPS is a condition of both malalignment and maltracking. The most widely accepted cause of PFPS is patellar maltracking, which occurs due to the weakness of the VMO [21]. This condition is also known as “runner’s knee”.

A conservative treatment of PFPS is focused on restoring standard patella tracking and strengthening the quadriceps muscle, which plays a vital role in maintaining the patella in its normal position. Patella taping was initially developed to create a mechanical medial shift to the patella, thereby centralizing it and improving patellar tracking. In the appropriate hands and with the right technique, patellar taping is successful [4], and it is very effective in reducing the level of pain during activities that create large patellofemoral joint reaction forces. Taping can also help reduce short-term pain with activity [22]. Additionally, a previous study showed that the strengthening the quadriceps, specifically the VMO, can impact the mechanics of the patellofemoral joint. The VMO is the only muscle that can medially move the patella and it works throughout the entire range of motion [23].

Moreover, past studies supported the use of electromyographic biofeedback (EMG-BF) in combination with conventional quadriceps exercises to improve symptoms and quadriceps strength in people with PFPS [24,25,26,27]. However, currently, there is no evidence for the effectiveness of EMG-BF as an addon to therapeutic patellar taping on pain, strength, or functional status in people with PFPS. Therefore, this study evaluated the effects of EMG-BF-guided isometric quadriceps strengthening with patellar taping and isometric exercise alone in young adult male athletes with PFPS to determine whether either of the two programs offered any advantages over the other for the rehabilitation protocol.

## 2. Materials and Methods

### 2.1. Study Design

**Figure 1:**
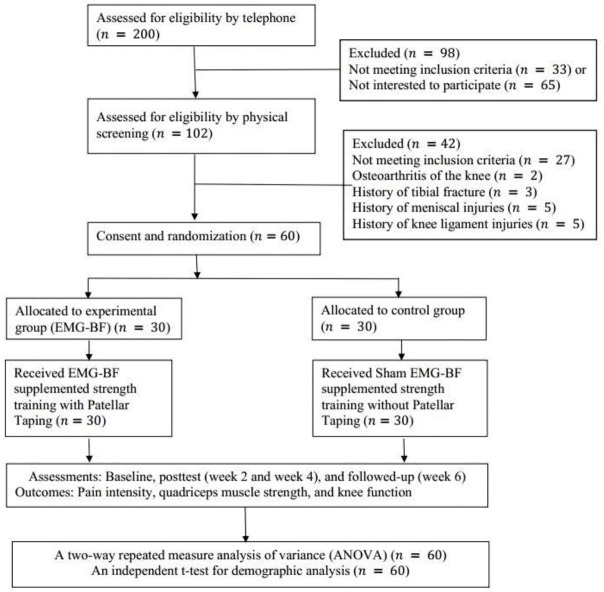
Consolidated Standards of Reporting Trials (CONSORT) diagram showing flow of participants through each stage of a randomized trial. EMG-BF, electromyographic biofeedback.

A randomized, controlled, single-blind study was designed to test the hypothesis, which comprised a 4-week intervention (as illustrated in Figure 1). The study was conducted between 30 November 2020 and 28 February 2021 at the physiotherapy and rehabilitation center at Majmaah University, Al Majma’ah, Riyadh, Saudi Arabia. Adult male athletes with PFPS were recruited through a local university, sporting clubs, and the community. They were screened by a senior physiotherapist who had more than 15 years of experience in the assessment and management of sport and musculoskeletal disorders. Pre- and post-test readings were taken at baseline, Weeks 2 and 4, and at the Week 6 follow-up. Random computer-generated numbers were used for the randomization process [28]. We used even and odd numbers to allocate an equal number of participants into the experimental and control groups [29]. Thirty participants in each group completed the trial. Group A (experimental group): EMG-BF-guided maximum voluntary isometric contraction (MVIC) exercise with patellar taping; Group B (control group): sham EMG-BF-guided MVIC without patellar taping. The outcome measure was the MVIC of the quadriceps muscle, pain intensity, and functional status.

### 2.2. Participants

A total of 200 university level athletes who had complained of knee pain were assessed via telephone. Sixty adult male athletes from two cities (Majmaah and Riyadh) in Saudi Arabia who met the inclusion criteria participated. The inclusion criteria were being a young male (between 18 to 35) with a history of knee pain during activities such as descending and ascending stairs, squatting, and running, and having a positive J sign (lateral tilt of the patella) and signs of patellar malalignment on a radiograph. More symptomatic and malaligned knees were included in the case of bilateral involvement. Individuals with a history of fractures around the knee, patellar dislocation, knee deformity (e.g., genu varum), flexion contracture, or ligament/meniscal injuries received NSAID or intra-articular injection, and those with osteoarthritis of the knee were excluded from the study. The ethical subcommittee of the College of Applied Medical Science, Majmaah, Saudi Arabia, approved this study (Ethics number: MUREC-Nov./COM-2O20/11-2). Under the Declaration of Helsinki, we informed participants about the study’s possible risks and benefits, and all participants signed a written consent form.

### 2.3. Outcomes

#### 2.3.1. Pain Intensity

The 10 cm visual analog scale (VAS) was used assess pain intensity. VAS is a reliable scale to measure knee pain [30,31]. Each participant was asked to report their current intensity of pain on a 10 cm scale between two anchors: 0 (indicating no pain) and 10 (indicating maximal pain).

#### 2.3.2. Knee Function

Knee function was assessed using the validated Arabic version of the Kujala Anterior Knee Pain scale [32,33]. It is a reliable questionnaire with which to assess PFPS in Arabic-speaking populations (Cronbach’s alpha, 0.824) [34]. It comprises 13 questions designed to evaluate difficulties related to PFPS, such as the presence of a limp, walking ability, need for support, squatting, stair climbing, jumping, running, pain, abnormal painful kneecap movements, prolonged sitting with knee flexed, atrophy of the thigh, swelling, and flexion deficiency. Score totals are in the range of 0–100. A higher score suggests lower symptoms and better functional capacity.

#### 2.3.3. Quadriceps Muscle Strength

The maximal voluntary isometric strength of the quadriceps femoris muscle was measured using a reliable and valid ISOMOVE dynamometer (ISO-MANSW-IT, Tecnobody, Dalmine, Bergamo, Italy). ISOMOVE system software version 0.0.1 (ISO-MANSW-IT, Tecnobody) was used to record all isometric data. It provides an accurate assessment of the maximal peak torque of quadriceps muscles. All subjects were familiarized with the equipment before the commencement of data collection.

**Figure 2:**
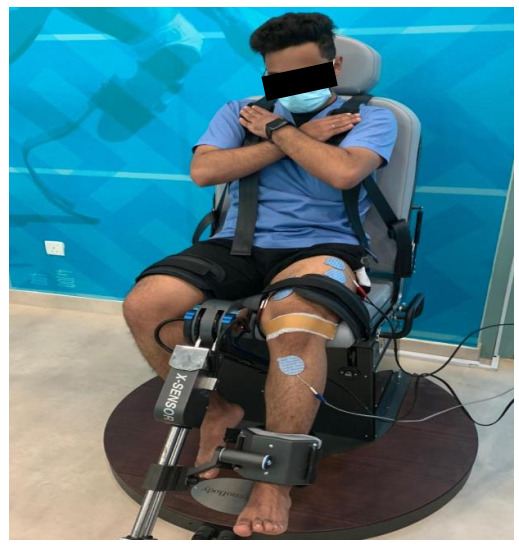
Measurement of maximal voluntary isometric contraction (MVIC) at 60° using ISOMOVE isokinetic dynamometer.

Participants were in a sitting position and stabilized with safety belts across the chest, thighs, and hips to avoid displacement; the shin pad was adjusted at 5.1 cm (2 inches) superior to the medial malleolus (as illustrated in Figure 2). Measurements were recorded from the participant’s most affected leg, with the hip and knee angles set at 60° of flexion, as this position resulted in the most significant torque output [22]. Each participant received verbal instruction to keep their arm crossed over his chest and verbal encouragement to motivate them to attain maximal effort during the 5 s contractions. Each test included 3 consecutive 5 s trials with a 2 min rest between the trials. The mean score of three readings was used for statistical analysis.

### 2.4. Interventions

#### 2.4.1. EMG-Biofeedback

Participants reclined in a supine position on a standard examination table and were guided with an EMG-BF two-channel device (Myomed 932, Enraf Nonius, Rotterdam, the Netherlands) to perform a strengthening protocol for the quadriceps muscles’ MVIC [35,36]. Participants were instructed to shave the area and wash with ethanol to decrease skin impedance before applying electrodes over the skin. Two pairs of disposable skin-adhesive surface electrodes were used to record muscle activity. A pair of electrodes were attached 3 cm medial and 4 cm superior to the superomedial aspect of the patella for VMO. Another pair of electrodes was attached at the lower third and middle of the thigh, slightly downward and medially for the rectus femoris (RF) muscle [35,36]. The interelectrode distance between the active electrodes was 2.1 cm, and electrodes were placed in each muscle’s fiber direction. A reference electrode was placed just below the tibial tubercle. Positions of the electrodes were marked by a permanent marker to make sure that the electrodes were placed over the same location across all sessions.

#### 2.4.2. Strength Training

1. Group A—Experimental group (EMG-BF-guided strength training along with patellar taping)

**Figure 3:**
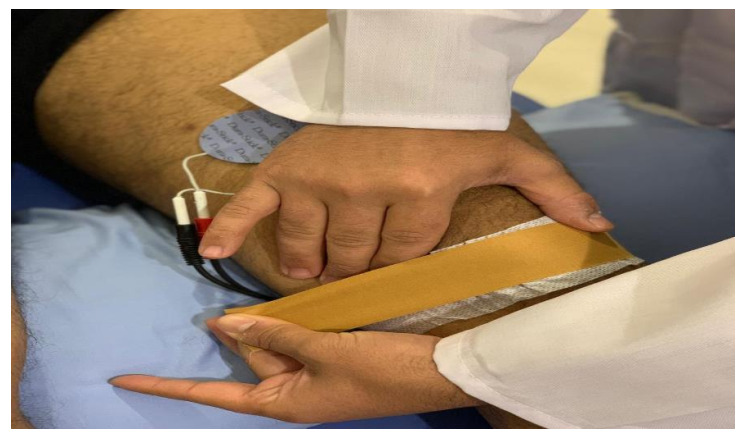
Application of McConnell taping.

In this group, participants received McConnell taping to correct the lateral and anteroposterior tilt and a medial patellar glide. It was applied distal to the patella to unload the infrapatellar fat pad (as illustrated in Figure 3). First, hypoallergenic underwrap tape was applied to prevent skin irritation; then, rigid (McConnell) tape was applied to maintain the patella by medially pulling the skin and patella [37]. Tape was removed after the outcome measure or if the participant experienced itching, redness, or discomfort.

Participants were given three sets of exercises to be performed five days a week for four weeks with patellar taping. The procedure was explained to the participants, and they were instructed to watch their muscle activity, increase VMO and RF activity levels during the exercises, hold the contraction for 5 s, and rest for 10 s. Before each session, the threshold level was identified by asking the participant to maximally contract the quadriceps muscle three times while an EMG-BF device monitored VMO and RF activity levels along with patellar taping. In the training session, the participants were instructed to voluntarily contract their VMO and RF above its threshold level and hold the contraction for 5 s to sustain the audible signal. Each participant performed three quadriceps-strengthening exercises such as isometric quadriceps, terminal knee extension, and isometric hip adduction exercises, as described below [35,36].

Isometric quadriceps exercise: participants were positioned in supine lying, and a roll of towel was placed below the knee. They were instructed to contract their quadriceps muscles above the threshold level to straighten the knees and to hold the contraction to maintain the audible signal for 5 s during the exercise for 3 sets of 10 repetitions each.

Terminal knee extension exercise: participants were positioned in sitting with the knee flexed from 60° to 0°. They were instructed to maximally activate their thigh muscles, contracting above the threshold level to straighten the knees, and to hold the contraction to maintain the audible signal for 5 s during the exercises for 3 sets of 10 repetitions each.

Isometric hip adduction exercise: participants were positioned in supine lying and a small towel was placed between the knees. They were instructed to perform an isometric hip adduction exercise by pressing the pillow between the knees and maximally activating the muscles, contracting above the threshold level to straighten the knees, and to hold the contraction to maintain the audible signal for 5 s during the exercises for three sets of 10 repetitions each.

2. Group B—Control group (sham EMG-BF-guided strength training without patellar taping)

**Figure 4:**
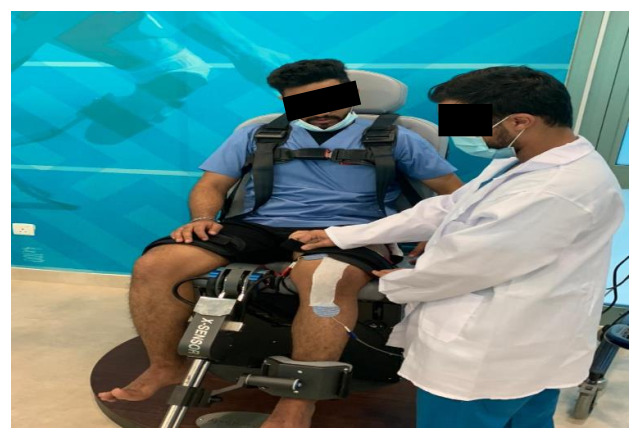
Application of nonrigid hypoallergenic tape (for placebo effect).

Subjects assigned to Group B were given the same set of exercises without EMG-BF. Nonrigid hypoallergenic tape (for a placebo effect) was applied on the skin in a vertical direction from the center of the patella while the participant was sitting with a flexed knee (as illustrated in Figure 4). However, electrodes were positioned away from the target muscles (e.g., VMO and RF). The reference electrode was positioned just below the tibial tuberosity. The participants received the placebo EMG-BF in which they performed quadriceps exercises with nonrigid hypoallergenic tape (placebo without rigid patellar taping) only, and they were not instructed to focus on the recruitment of VMO and RF muscle.

### 2.5. Statistical Analysis

Data were analyzed using Statistical Programming for Social Studies (SPSS) software for Windows (IBM Corp. version 22, Armonk, NY, USA). Demographic data including age, height, weight, and body-mass index were compared using an independent t-test. Data normality was confirmed by Shapiro–Wilk test (*p* > 0.05). A two-way (2×4) repeated measure analysis of variance (ANOVA) with Tukey’s multiple-comparisons test was conducted to compare pain intensity, quadriceps muscle strength, and knee function between the two groups. The two factors were treatment group (two levels) and time (four levels). In addition, the effect size was calculated using Cohen’s d (d = <0.5, small; d = 0.50 to 0.80, medium; and d >0.80, large) to assess changes in all outcomes between the two groups. All data were considered to be statistically significant if *p* < 0.05.

## 3. Results

Table 1 details the demographic statistics. Results of the demographic data revealed insignificant differences between the two groups (All *p* > 0.05). The comparison of VAS score between two groups is given in Table 2. The experimental group demonstrated a significantly lower VAS score at Weeks 2 and 4 compared to that of the control group (*p* = 0.008 and 0.0005, respectively). It remained significantly lower in the experimental group after follow-up at Week 6 compared to the control group (*p* = 0.0005).

Table 3 presents the comparison of quadriceps muscle strength in the two groups. There were no differences in quadriceps strength at Week 2 between the two groups (*p* = 0.259). However, the experimental group demonstrated significantly higher quadriceps strength at Weeks 4 and 6 compared to the control group (*p* = 0.0008).

Table 4 illustrates the comparison of knee function between the two groups. There were no differences in knee function at Weeks 2 and 4 between the two groups (*p* = 0.086 and 0.171, respectively). However, the experimental group showed significantly better knee function at Week 6 compared to that of the control group (*p* = 0.002).

## 4. Discussion

This study evaluated the effects of EMG-BF-guided isometric quadriceps strengthening with patellar taping and isometric exercise alone in young adult male athletes with PFPS. The results of this study revealed significantly better outcomes in terms of pain intensity and quadriceps muscle strength after four weeks of EMG-BF-guided isometric quadriceps strengthening with patellar taping in people with PFPS. While there was an insignificant difference in knee function between the experimental and control groups over the four weeks of intervention, the experimental group showed better knee function in the follow-up at Week 6.

EMG-BF is a physiotherapeutic technique that is often used with other exercise programs such as strength training to reduce pain and improve muscle strength in people with PFPS and knee osteoarthritis [26,35,36,37,38,39]. EMG-BF-guided strength training encourages active participation, which motivates patients to play an active role during the training period in achieving their desired therapeutic rehabilitation goals [37]. EMG-BF-guided strength training is conducted by a special type of biofeedback device that records muscle tone and tension measured in microvolts via the application of surface or needle electrodes [40,41]. Patients can see changes in muscle tension (increase or decrease) in the form of auditory or visual feedback. Therefore, patients can control and modify their muscle tension with the help of a biofeedback device [42].

Many past studies used EMG-BF-guided strength training in the management of symptomatic PFPS [24,25]. For instance, Wise et al. [24] piloted a study to examine the effects of an EMG-BF-guided progressive exercise program in people with PFPS. They suggested that the EMG-BF, along with a graded exercise program, is an effective and efficient therapeutic approach for managing symptoms in people with PFPS. Additionally, Ng et al. [25] concluded that the application of an EMG-BF-guided exercise program was effective in improving the VMO/vastus medialis longus EMG ratio in people with PFPS. In contrast, other studies reported no additional therapeutic benefits of EMG-BF compared with quadriceps exercise alone in people with PFPS [26,42,43,44].

This study is the first to evaluate the combined effects of EMG-BF and patellar taping in people with PFPS. While the EMG-BF group demonstrated better improvement compared to the control group, it was difficult to assess the isolated effects of patellar taping on pain intensity or quadriceps muscle strength in people with PFPS. Several research studies that evaluated the application of patellar taping showed potential benefits for perceived pain reduction in people with PFPS [14,15,16]. A few studies found that the application of patellar taping over the skin can improve proprioception by activating cutaneous mechanoreceptors, thereby enhancing afferent input to the central nervous system (CNS) [17,18]. This outcome is known as the nociceptive effect, which facilitates large afferent fiber input and elicits neural inhibition [14,17,18]. In contrast, other studies reported that the application of patellar taping demonstrated no additional benefit to the quadriceps strengthening exercise, as the application of patellar taping neither decreased pain nor increased force production in people with PFPS [20,21].

Our study has some limitations. First, since female participation in sport in Saudi Arabia is relatively restricted compared to that of males [45], it was difficult to recruit female athletes with PFPS. Thus, this study was limited to only male athletes with PFPS. Therefore, the results of this study cannot be generalized to female athletes with PFPS. Second, the intervention effects in this study were followed up for only two weeks. Therefore, future studies are needed to examine the long-term effects of EMG-BF-guided strength training in people with PFPS. Lastly, the isolated effect of EMG-BF-guided strength training and patellar taping was not evaluated. Therefore, further studies are warranted to investigate whether the application of patellar taping could play an additive role to augment the therapeutic effect of EMG-BF in people with PFPS.

While EMG-BF is used frequently in many medical conditions such as headache and stroke, the evidence for its application in sports and musculoskeletal disorders including PFPS is limited. The current study suggested that the use of EMG-BF with patellar taping could be a potential therapeutic modality to reduce pain, and improve muscle strength and function in the rehabilitation of athletes with PFPS. Additionally, strength training with EMG-BF along with patellar taping is feasible, responsive, and could maximize the recovery potential in the rehabilitation of PFPS in young athletes. Moreover, it helps to initiate early, vigorous training if there is a precaution for performing dynamic strength techniques.

## 5. Conclusions

This study examined the combined effects of EMG-BF-guided isometric quadriceps strengthening and patellar taping compared with an isometric exercise alone in PFPS among young adult male athletes. Four weeks of EMG-BF supplementation training with patellar taping demonstrated significantly better improvement in pain intensity, functional disability, and quadriceps muscle strength in young adult male athletes with PFPS. Future longitudinal studies are warranted to examine the long-term effects of these interventions in people with PFPS.

## Figures and Tables

**Table 1:** Demographic statistics.

Variables	EMG-BF (*n* = 30) Mean ± SD	Control (*n* = 30) Mean ± SD	* *t*-test	Effect Size (Cohen’s d)
			*p* Value	
Age, years	25.9 ± 1.5	26.8 ± 2.2	0.087	0.48
Height, cm	167.6 ± 2.5	168.5 ± 3.2	0.235	0.31
Weight, kg	66.5 ± 3.9	67.8 ± 4.2	0.247	0.32
BMI, kg/m^2^	23.8 ± 0.9	24.1 ± 0.8	0.322	0.35

* Statistically significant if *p* < 0.05; EMG-BF, electromyographic biofeedback; effect size: small (0.20), medium (0.50), large (0.80).

**Table 2:** Comparison of visual analog scale (VAS) scores between the two groups.

VAS (0–10 cm)	EMG-BF (*n* = 30) Mean ± SD	Control (*n* = 30) Mean ± SD	* ANOVA		Effect Size (Cohen’s d)
			F	*p*	
Baseline	6.95 ± 0.66	6.76 ± 0.69	1.30	0.408	0.28
Week 2	3.75 ± 0.60	5.1 ± 1.1	40.68	0.0008	1.65
Week 4	1.68 ± 0.67	4.45 ± 0.86	19.74	0.0005	1.15
Week 6	1.25 ± 0.78	4.03 ± 0.61	19.86	0.0005	1.15

* Statistically significant if *p* < 0.05; EMG-BF, electromyographic biofeedback; effect size: small (0.20), medium (0.50), large (0.80).

**Table 3:** Comparison of quadriceps strength between two groups.

Strength (in kg)	EMG-BF (*n* = 30) Mean ± SD	Control (*n* = 30) Mean ± SD	* ANOVA		Effect Size (Cohen’s d)
			F	*p*	
Baseline	11.47 ± 1.34	11.97 ± 1.11	2.49	0.178	0.41
Week 2	12.77 ± 1.19	12.44 ± 1.06	1.64	0.259	0.33
Week 4	14.06 ± 1.03	13.18 ± 1.08	4.37	0.0008	0.54
Week 6	15.25 ± 0.89	14.14 ± 1.07	5.50	0.0008	0.61

* Statistically significant if *p* < 0.05; EMG-BF, electromyographic biofeedback; effect size: small (0.20), medium (0.50), large (0.80).

**Table 4:** Comparison of knee function between two groups.

Function (0–100)	EMG-BF (*n* = 30) Mean ± SD	Control (*n* = 30) Mean ± SD	* ANOVA		Effect Size (Cohen’s d)
			F	*p*	
Baseline	42.56 ± 6.64	46.1 ± 7.74	2.76	0.266	0.43
Week 2	63.86 ± 9.42	60.8 ± 7.29	2.34	0.086	0.40
Week 4	73.26 ± 7.22	69.06 ± 6.04	3.28	0.171	0.47
Week 6	80.36 ± 5.01	72.5 ± 5.99	6.15	0.0002	0.64

* Statistically significant if *p* < 0.05; EMG-BF, electromyographic biofeedback; effect size: small (0.20), medium (0.50), large (0.80).

## Data Availability

All datasets related to the results of this study are available from the primary author on request.
